# Combined Operations for the Cure of Procidentia Uteri

**Published:** 1890-06

**Authors:** Henry C. Coe

**Affiliations:** Professor of Gynæcology at the New York Polyclinic


### COMBINED OPERATIONS FOR THE CURE OF PROCIDENTIA UTERI.

BY HENRY C. COE, M. D., M. R. C. S.,
*Professor of Gynæcology at the New York Polyclinic.*

This patient, whom you had an opportunity to examine at the Polyclinic last week, is 50 years of age, and has not menstruated for five or six years. She has had complete procidentia for eight years, and has worn pessaries with some relief, but now desires to be radically relieved by an operation if possible. There is every reason why the uterus should be kept within the vagina aside from the personal inconvenience to the patient of the present displacement. You see that there is a bad erosion of the cervix, due to the constant chafing of the part against the thighs; this prolonged irritation might in time give rise to cancer, as in a striking case which I saw in the Middlesex Hospital in London. As regards the choice of operation, there comes first in the order of its seriousness extirpation of the procident uterus per vaginam. I performed this operation successfully last fall, but only as a last resort, in the case of a patient who had undergone six unsuccessful operations before, including shortening of the round ligaments; I would certainly not feel justified in doing it in the present instance. Next comes ventro-fixation, or opening the abdomen, replacing the uterus and endeavoring to keep it in its normal position by shortening the round ligaments within the pelvic cavity, or attaching the organ to the abdominal wall. This is also too radical to fit the present case. Next in order is Alexander's operation, one which I would not expect to give permanent relief in the present case, because the uterus is so large and heavy and the round ligaments are doubtless atrophied; even an additional perineal operation would not supply the necessary support. A combined anterior and posterior colporrhaphy seem to me to be equally uncertain in their results. Since this patient has passed the menopause, I propose to perform Le Fort's operation, or close the vagina completely by denuding its anterior and posterior walls and bringing them in contact, thus affording a solid fleshy column on which the uterus can rest. Before doing this I shall excise the cervix and bring the edges together with catgut sutures, since I am afraid to close in such a red, angry-looking surface which may give trouble in the future when it can not be got at so easily. This operation is very simple, since the cervix lies right before me without the necessity of using a speculum. I am careful, as you see, to leave a wide canal in the centre to provide for the escape of secretions, since it is impossible under the circumstances to use a plug. I shall have this eroded tissue sent to the pathologist for a careful report, as I do in every instance, because I am deeply interested in tracing the relation between cervical erosions and the development of epithelioma. Notice that this cervix looks as if it had never been lacerated, the erosion is due purely to the prolonged mechanical irritation. Having closed the cervix, I now draw it strongly downward and denude a large parallelogram on the anterior vaginal wall, extending from the anterior fornix down to a point only half an inch above the meatus. There is hardly any hemorrhage, from the fact that the entire prolapsed mass is tightly grasped by the vulva, which acts as a perfect tourniquet. The cervix is next drawn well upward, and a similar surface is denuded on the posterior vaginal wall.

Le Fort's original directions do not include the introduction of sutures through the middle of the wound, but only at its lower transverse and lateral borders. It seems to me that a much better and more permanent result is obtained by introducing several rows of buried catgut sutures in the middle of the wound, each row closing in the preceding one; I have never seen any one else do this operation, but this is my habitual practice. I enter a curved needle threaded with No 2 catgut close to the right lateral edge of the denuded surface on the anterior vaginal wall at a point a quarter of an inch above its lower (really upper) transverse border, and bring it out close to the latter; then drawing the cervix upward, I reverse the procedure, entering it near the lower edge and coming out in the raw surface close to the lateral border, so that when the suture is pulled through it crosses over the os externum. Four sutures are thus introduced. Now my assistant pushes up the cervix with a sponge-holder and I tie the sutures. The cervix, as you see, has disappeared; it has been inverted just as you would invert a glove-finger by indenting it on the end with your finger. This process of inversion is to be continued until the entire raw surfaces have been rolled in, *i. e.*, until the uterus has been replaced.

This procedure is long and tedious, since at least 20 sutures must be used, but you will admit that it insures good union. I put an occasional suture in the lateral border to give greater solidity to the barrier which I am erecting against the downward pressure of the heavy uterus. The denuded surfaces have now been rolled in and brought in close opposition down to their lower transverse borders, through which I pass an additional row of sutures to support the last row. The vagina has been practically closed, but a canal exists at each side, communicating with the hollow at the upper end of the supporting column, in which rests the cervix. Provision is thus made for the escape of discharges from the uterus.

To afford additional support I shall do a Tait's perinæum, which is preferable in the present case, not only because there is really no external tear, only an overstretching of the parts from long distention of the vulva, but because the operation requires only a few minutes and the patient has already been under ether over an hour. With two fingers introduced into the rectum, I introduce these short angular scissors at a point to the left of the vulva and just at its edge, and cut straight across to a corresponding point on the opposite side. With a few snips a hole is made in the perineal body between the rectum and vagina, the vaginal and rectal flaps are forced by two short lateral incisions extending obliquely outward from the ends of the transverse one, and are drawn apart by tenacula. We have now a diamond-shaped denuded surface, and have only to bring the opposite borders together, which is easily done by four silk-worm gut sutures, threaded on common round-pointed sewing needles. As you see, I have not removed my fingers from the rectum since I began the operation, which is completed in ten minutes. I can not speak so enthusiastically of this as of other operations for the repair of lacerated perinæum, for although it is ingenious, easy of execution, and almost invariably successful as regards primary healing, I have not found, after the lapse of several weeks or months, that solid perineal body which alone we seek to obtain by this operation. The flap-splitting operation is a most admirable one for impressing spectators with the operator's rapidity and dexterity, but to operate "against time" should never be the ambition of the conservative surgeon. I have come to the conclusion that I shall do the operation only in cases like the present, in which it is intended merely to supplement preceding operations, which have already kept the patient sufficiently long under ether.

A pad of iodoform gauze is applied to the vulva and is secured by a firm T-bandage. The patient's urine will be drawn for the first few days in order to prevent straining. Her bowels will be kept loose for the same reason.

[The perineal sutures were removed on the eighth day, and the patient was allowed to sit up on the twelfth, still wearing the T-bandage, union of both wounds being good.—[*Annals of Gynaecology and Pediatry.*

